# Efficacy and safety of sedation with dexmedetomidine in adults undergoing gastrointestinal endoscopic procedures: systematic review and meta-analysis of randomized controlled trials

**DOI:** 10.3389/fphar.2023.1241714

**Published:** 2023-11-15

**Authors:** Rou Tang, Yaqun Huang, Yujia Zhang, Xiaolei Ma, Haoyang Yu, Kaichao Song, Ling Ren, Bin Zhao, Lulu Wang, Wensheng Zheng

**Affiliations:** ^1^ Institute of Materia Medica, Chinese Academy of Medical Sciences and Peking Union Medical College, Beijing, China; ^2^ Department of Pharmacy, Peking Union Medical College Hospital, Beijing, China; ^3^ Department of Pharmacy, Hospital of Honghe State Affiliated to Kunming Medical University, Southern Central Hospital of Yunnan Province, Mengzi, China; ^4^ Institute of Medicinal Biotechnology, Chinese Academy of Medical Science and Peking Union Medical College, Beijing, China

**Keywords:** dexmedetomidine, gastrointestinal endoscopic procedures, sedative, metaanalysis, randomized controlled trial

## Abstract

**Background:** The sedative role of dexmedetomidine (DEX) in gastrointestinal endoscopic procedures is unclear. We performed this systematic review and meta-analysis to assess the efficacy and safety of sedation with DEX during gastrointestinal endoscopic procedures with a view to providing evidence-based references for clinical decision-making.

**Methods:** The PubMed, Embase, Cochrane Library, Web of Science, and ClinicalTrials.gov databases were searched for randomized controlled trials (RCTs) that compared DEX with different sedatives comparators (such as propofol, midazolam, and ketamine) for sedation in a variety of adult gastrointestinal endoscopic procedures from inception to 1 July 2022. Standardized mean difference (SMD) and weighted mean difference (WMD) with 95% confidence interval (CI) or pooled risk ratios (RR) with 95% CI were used for continuous outcomes or dichotomous outcomes, respectively, and a random-effect model was selected regardless of the significance of the heterogeneity.

**Results:** Forty studies with 2,955 patients were assessed, of which 1,333 patients were in the DEX group and 1,622 patients were in the control (without DEX) group. The results suggested that the primary outcomes of sedation level of DEX are comparable to other sedatives, with similar RSS score and patient satisfaction level, and better in some clinical outcomes, with a reduced risk of body movements or gagging (RR: 0.60; 95% CI: 0.37 to 0.97; *p* = 0.04; I^2^ = 68%), and a reduced additional requirement for other sedatives, and increased endoscopist satisfaction level (SMD: 0.41; 95% CI: 0.05 to 0.77; *p* = 0.03; I^2^ = 86%). In terms of secondary outcomes of adverse events, DEX may benefit patients in some clinical outcomes, with a reduced risk of hypoxia (RR:0.34; 95% CI: 0.20 to 0.55; *p* < 0.0001; I^2^ = 52%) and cough (RR: 0.25; 95% CI: 0.12 to 0.54; *p* = 0.0004; I^2^ = 0%), no significant difference in the risk of hypotension, while an increased risk of bradycardia (RR: 3.08; 95% CI: 2.12 to 4.48; *p* < 0.00001; I^2^ = 6%).

**Conclusion:** This meta-analysis indicates that DEX is a safe and effective sedative agent for gastrointestinal endoscopy because of its benefits for patients in some clinical outcomes. Remarkably, DEX is comparable to midazolam and propofol in terms of sedation level. In conclusion, DEX provides an additional option in sedation for gastrointestinal endoscopic procedures.

**Systematic Review Registration:** https://www.crd.york.ac.uk/PROSPERO/#searchadvanced

## Introduction

Conscious sedation is a common strategy to increase patient comfort during gastrointestinal endoscopy, as it is an uncomfortable and stressful procedure for most patients. It improves clinical outcomes by decreasing procedural pain, increasing patient satisfaction, relieving patient anxiety and discomfort, and minimizes the risk of adverse effects by avoiding involuntary and untimely patient movements that might interfere with endoscopic procedures (Early et al., 2018; Kamal et al., 2021; Mason et al., 2021). A number of agents are available for conscious sedation during endoscopic procedures, including benzodiazepines (midazolam), opioids (fentanyl and meperidine), propofol, and dexmedetomidine (DEX) (Waring et al., 2003; Vargo et al., 2012).

Dexmedetomidine (DEX) is a potent, highly selective α2-adrenergic receptor agonist with the properties of sedation, analgesia, anxiolysis, and sympathetic tone inhibition (Aantaa and Scheinin, 1993; Kamibayashi and Maze, 2000). DEX was first approved for sedation in intensive care units (ICU) in 1999, and its use has been rapidly extended to patients sedation in a variety of clinical situations (Takrouri et al., 2002). DEX has recently gained popularity in gastrointestinal endoscopic procedures due to its superiority over conventional sedatives, including cooperative or semi-rousable sedation, minimal respiratory depression at high doses, and fewer cardiopulmonary complications (Ebert et al., 2000; Venn et al., 2000; Liu X et al., 2021). However, associated adverse effects of DEX such as hypotension, the biphasic dose-response relationship of mean arterial pressure, and bradycardia have been reported (Ebert et al., 2000; Talke et al., 2003; Bharati et al., 2011). Despite the widespread use of DEX, significant concerns remain about its safety and efficacy. Several meta-analyses of randomized controlled trials (RCTs) and many clinical studies have compared DEX with other conventional sedatives (Nishizawa et al., 2015; Zhang et al., 2016; Nishizawa et al., 2017; Liu W et al., 2021), due to the limited number of studies and the single perspective (simply comparing DEX with a particular sedative), no clear conclusions have been drawn about the role of dexmedetomidine for sedation in gastrointestinal endoscopic procedures. There are even some conflicting conclusions, for instance, a previous study reported that DEX is associated with better sedation than midazolam in gastrointestinal endoscopic procedures, however, another study showed that DEX may be a possible alternative to midazolam in sedation (Nishizawa et al., 2015; Zhang et al., 2016).

The many favorable physiological effects of DEX have made it increasingly popular and applied in gastrointestinal endoscopic procedures, however, no current literature reviews are providing a definite conclusion about the role of DEX in gastrointestinal endoscopic procedures sedation. We, therefore, performed an updated, systematic, and pooled meta-analysis of the currently associated RCTs to evaluate the efficacy and safety of DEX compared with multiple conventional sedatives in various gastrointestinal endoscopic procedures.

## Methods

### Data sources and literature search strategy

We conducted this meta-analysis in accordance with the Preferred Reporting Items for Systematic Review and Meta-Analysis (PRISMA) guideline (Higgins et al., 2011). It was prospectively registered on PROSPERO (CRD42022345358). We performed a comprehensive search of the MEDLINE (via PubMed), Embase (via Ovid), Cochrane Central Register of Controlled Trials (CENTRAL), Institute for Scientific Information (ISI) Web of Science, and the ClinicalTrials.gov database from inception to 1 July 2022. The search comprised free-text terms and database-specific subject headings for DEX in combination with endoscopic procedures. The full search strategies for all databases are provided in Supplementary Material SA. The PRISMA checklist is provided in SI B.

Two authors (RT and YH) independently screened the titles and abstracts of all studies retrieved by the search strategy. We excluded obviously irrelevant studies and documented the reason for exclusion of studies when the reason for exclusion is not explicit. Eligibility for the remaining studies then be identified by reading the full text and according to predefined inclusion and exclusion criteria, besides, we reviewed the reference lists of all eligible studies and previously published systematic reviews and meta-analyses to identify additional relevant randomized controlled trials. We attempted to contact the first author of the relevant trial when further information was required or any queries arose. We resolved disagreements between the two authors (RT and YH) by discussion until consensus is reached or by consulting with a third author (YZ).

### Inclusion and exclusion criteria

We included studies meeting the following inclusion criteria: 1) study type: RCT; 2) population: adult patients (16 years of age or older) undergoing all types of diagnostic and therapeutic gastrointestinal endoscopic procedures; 3) intervention: perioperative administration of DEX alone or in combination, irrespective of the route of administration, dosage, frequency, and duration; 4) comparator: any other pharmacological interventions, including other sedative agents such as propofol, midazolam, and ketamine, or 0.9% sodium chloride, or placebo; 5) outcomes: eligible studies had to report at least one of the predetermined outcomes listed in the following: a) primary outcomes: sedation level (Ramsay sedation scale (RSS) score, body movements or gagging, endoscopist and patient satisfaction level, and reduction in other sedative requirements); b) secondary outcomes: adverse events (hypoxia, hypotension, bradycardia, and cough). Only full articles published in English were considered.

Duplicate publications, reviews, prospective cohort studies, cross-over trials, quasi-randomized trials, and all nonrandomized trials were excluded.

### Data extraction

Four authors (RT and YH; XM and HY; in pairs) independently performed data extraction using predesigned data extraction forms. We extracted the following characteristics from each included study: the first author, year of publication, country or location where the study was conducted, study quality, sample size, details of participants (such as age and sex), inclusion criteria, exclusion criteria, type of endoscopic procedure, details of intervention (such as route of administration, dosage, and duration of DEX, and comparator medication), outcomes (as listed in above), adverse events, and risk of bias. When studies reported multiple treatment arms using additional sedatives, only data from the groups utilizing DEX were extracted. Data reported in graph form were extracted by the software GetData Graph Digitizer (v2.25, Canopus, Japan).

Any discrepancies in extracted data were resolved by a repeat review of the original text and discussion with a third author (KS and LR).

### Risk of bias assessment

Two authors (YZ and KS) independently assessed risk of bias for each included study using the Cochrane risk of bias tool described in the Cochrane Collaboration Handbook for Systematic Reviews of Interventions (Higgins et al., 2011). Included studies were assessed at low, high, or unclear risk of bias across seven domains applicable to RCTs: random sequence generation, allocation concealment, blinding methods, blinding of participants and personnel, blinding of outcome assessment, incomplete outcome data, selective outcome reporting, and other sources of bias. Any disagreements were resolved by a repeat review of the data and consensus through discussion, or arbitration by a third author if necessary.

### Data synthesis and statistical analysis

We calculated the standardized mean difference (SMD) and weighted mean difference (WMD) with 95% confidence interval (CI) or pooled risk ratios (RRs) with 95% CI for continuous outcomes or dichotomous outcomes, respectively; for other outcomes, we performed a qualitative analysis. Measurement data presented as mean (SE) or mean will be excluded. For the purposes of this review, we included the studies reporting the range or inter-quartile range (IQR), and standard deviation (SD) was estimated with the formulas: $mean=\frac{a+2m+b}{4}$ and $SD=\frac{b-a}{2ø-1\left(\frac{n-0.375}{n+0.25}\right)}$ , or $mean=\frac{a+2q1+2m+2q3+b}{8}$ and $SD=\frac{b-a}{4ø-1\left(\frac{n-0.375}{n+0.25}\right)}+\frac{q3-q1}{4ø-1\left(\frac{0.75n-0.125}{n+0.25}\right)}$ , respectively, which estimated the sample mean and SD from the sample size, median, range and/or IQR (Wan et al., 2014; Luo et al., 2018). The median was used to estimate the mean if a value for the mean was not provided.

The heterogeneity across studies was assessed with Cochran’s Q test (*p* < 0.10 for statistical significance) and the I^2^ statistic (I^2^ > 50% for significant heterogeneity). We always used a random-effect model, regardless of the significance of the heterogeneity. Regardless of the level of heterogeneity (significant or not), we performed subgroup analyses to explore possible sources of clinical heterogeneity or to assess the effect of grouping factors on outcomes: 1) different comparators (saline or other sedative agents such as propofol, midazolam, and ketamine, or opioids (including fentanyl, sufentanil, remifentanil, and meperidine)); 2) surgery type that is divided into two groups based on the procedure length (the non-advanced endoscopic procedures consisting of gastroscopy, colonoscopy, gastrointestinal endoscopy, diagnostic esophagogastroduodenal endoscopy, and esophagogastroduodenoscopy, and the advanced endoscopic procedures including endoscopic retrograde cholangiopancreatography (ERCP), and endoscopic submucosal dissection (ESD) ESD; 3) different scoring systems or definitions for some outcomes (a. different scoring systems for endoscopist satisfaction level (the numeric rating scores (1–4), or the visual analogue scale (VAS) scores (0–10 or 0–100)); b. different scoring systems for patient satisfaction level (the seven-step numeric range Likert scale (1–7), or the VAS scores (0–10 or 0–100)); c. different definitions for hypoxia (SpO_2_ < 90% or <94%)). Subgroup analysis was performed only if there were at least two studies in each subgroup, and the data were analysed by χ^2^ test.

Additionally, sensitivity analyses were performed to assess the effect of individual studies with a high risk of bias on the stability of pooled data. Finally, publication bias was detected by funnel plot asymmetry with Egger’s regression tests. All statistical analyses will be performed using Review Manager 5.4. (RevMan, v5.4, The Cochrane Collaboration, Oxford, UK) and Stata/SE 17.0 (Stata Corp., TX, United States).

## Results

### Literature search results and study characteristics

**FIGURE 1:**
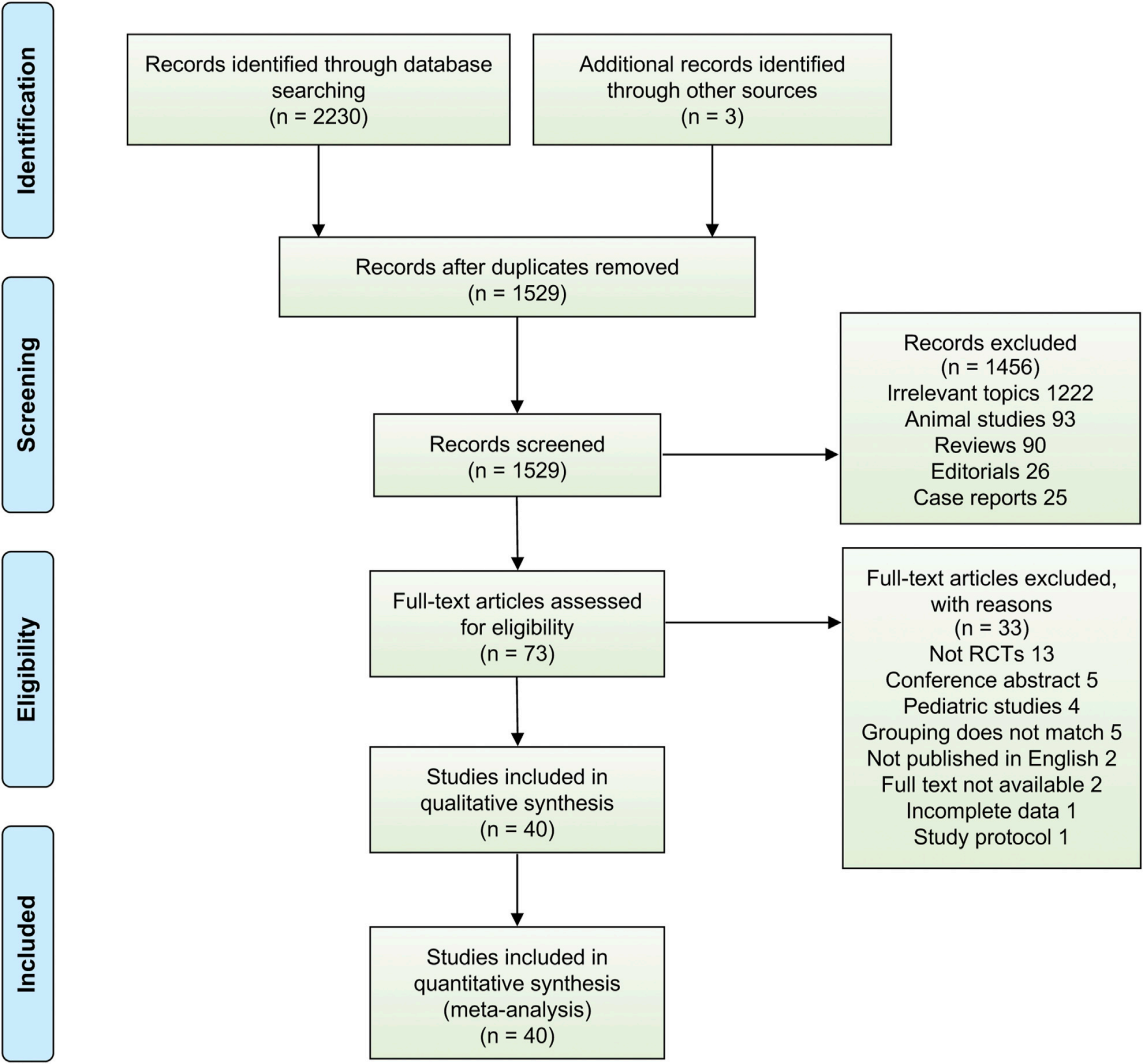
PRISMA flowchart of database search and study identification.

Using the aforementioned literature search strategy, 2,233 potentially relevant citations were found through a systematic search, and 1,529 articles remained after exclusion of duplicates. Of those, 1,456 citations were removed after title and abstract screening, 73 studies underwent a full-text review, and 33 studies were subsequently excluded after the full-text review. Finally, we included 40 RCTs that fulfilled the eligibility criteria for analysis, and a detailed overview of PRISMA flowchart of database search and study identification is shown in Figure 1, reflecting the search process and the reasons for exclusion.

The characteristics of included trials are summarized in Table 1. 40 RCTs enrolling a total of 2,955 patients who underwent gastrointestinal endoscopic procedures were included in this meta-analysis. Of those, 1,333 patients were in the DEX group (alone or in combination) and 1,622 patients were in the control (without DEX) group. We pooled saline and other sedatives as a collective control group. Of these included trials, 18 studies used DEX alone, whilst 22 studies used DEX in combination with other sedatives; 31 studies involved one DEX intervention group and one control group, whilst 9 studies set ≥2 control groups; 8 studies compared DEX with saline, whilst 32 studies included other comparators (propofol, midazolam, ketamine, fentanyl, remifentanil, sufentanil, and meperidine). Nineteen studies were non-advanced endoscopic procedures, and the other 21 studies were advanced endoscopic procedures.

**TABLE 1:** Characteristics of included studies.

Study	Sample size	Intervention study (DEX dose/administration mode)	Sedatives	Surgery type	ASA	Rescue drug	Outcome measures
	DEX/Control						
Cheung et al. (2015)	25/25	Intranasally (1.5 μg/kg) 1 h before the procedure	DEX	EGD	I–II	PF and alfentanil	5, 6
			Saline				
Yin et al. (2019)	30/30/30/30	Infused (0.1 mL/kg, 4 μg/mL) over 5 min	PF + DEX	EGD	I–III	PF	1, 2, 5–8, 9
			PF + Saline				
			PF + Sufentanil				
			PF + Ketamine				
Eberl et al. (2016)	31/31	Bolus loading dose (1 μg/kg (0.5 μg/kg in patients >65 years)) over 10 min, and then (0.7–1 μg/kg/h) maintaining during procedure	DEX	EOPD	I–III	PF or alfentanil	3–5
			PF				
Edokpolo et al. (2019)	51/50	Bolus dose of 0.3 μg/kg	PF + DEX	CS	I–III	PF	10
			PF + Saline				
Goyal et al. (2016)	41/42	Bolus (0.5 μg/kg) over 30 s, and then (0.5 μg/kg/h) during procedure	DEX + Ketamine	ERCP	I–III	Ketamine or PF	2, 6–8
			PF + Fentanyl				
Ashikari et al. (2021)	33/33	Bolus (6 μg/kg/h) at first 10 min, and then (0.7 μg/kg/h) for maintenance	PF + DEX	ESD	I–II	PF	3, 5, 6–8
			PF + Saline				
Wu et al. (2014)	30/30	Bolus (0.3 μg/kg) 10 min before endoscopy, and then (0.2–0.3 μg/kg/h) continuous infusion	Fentanyl + DEX	EGD	I–II	MDZ or fentanyl	1,
			Fentanyl + MDZ				
Mazanikov et al. (2013)	25/25	Loading dose (1 μg/kg) infused over 10 min, and then (0.7 μg/kg/h) continuous intravenous infusion during procedure	DEX	ERCP	I–III	PF and alfentanil	4, 5, 6, 7
			DEX + Saline				
Kinugasa et al. (2018)	40/40	Loading does (6.0 μg/kg/h) infusion for 5 min, then maintained at 0.4 μg/kg/h (increasing or decreasing by 0.1 μg/kg/h)	DEX	ESD	N.A.	Pethidine	3–5, 6–8
			Placebo				
Lu et al. (2018)	108/86	Bolus (1 μg/kg (0.5 μg/kg in patients >65 years)) over 10 min	Remifentanil + DEX	ERCP	I–III	Remifentanil and MDZ	1–5, 6
			Remifentanil + MDZ				
Demiraran et al. (2007)	25/25	Intravenous infusion (1 μg/kg) over 10 min before procedure, and then continuous infusion (0.2 μg/kg/h)	DEX	ESOD	I–II	N.A.	3, 4, 6, 8, 9
			MDZ				
Akarsu Ayazoğlu et al. (2013)	30/30/31/30	Infusion (0.2 μg/kg/h) during procedure	PF + DEX	CS	I–II	N.A.	5
			PF + Sufentanil				
			PF + Meperidine				
			PF + Meperidine + MDZ				
Aminnejad et al. (2022)	33/31	Intravenously infusion (0.3 μg/kg)	DEX + Ketamine	CS	I–II	N.A.	1, 7, 8
			PF + Fentanyl				
Vázquez-Reta et al. (2011)	20/20	Loading dose (1 μg/kg) infusion over 20 min, and then maintaining infusion (0.2 μg/kg/h)	DEX	EGD	I–II	N.A.	1, 4, 7, 8
			MDZ				
Wu et al. (2015)	33/34	Loading dose (1 μg/kg) over 10 min, and then maintaining infusion (0.5 μg/kg/h)	DEX	ESOD	I–II	PF	2–4, 6, 8
			PF				
Chen et al. (2022)	24/25	Loading dose intravenous (0.5 μg/kg) for 10 min at the start of anesthesia induction	DEX	ERCP	I -III	N.A.	7, 8
			PF				
Kim et al. (2015)	29/30	Bolus (0.5 μg/kg) 5 min before starting procedure, and then continuous infusion (0.3–0.7 μg/kg)	Remifentanil + DEX	ESD	I-III	PF and remifentanil	5
			Remifentanil + PF				
Lee et al. (2014)	53/57	Continuous I.V. infusion (1 μg/kg/h)	MDZ + Meperidine + DEX	ERCP	I-III	MDZ and meperidine	1, 5, 6–8
			MDZ + Meperidine + Saline				
Takimoto et al. (2011)	30/30/30	I.V. injection for 5 min at (3 mg/kg/h) for introduction, and then I.V. at (0.4 mg/kg/h) for maintenance	DEX + Pentazocine	ESD	N.A.	MDZ	2, 5, 6, 7
			PF + Pentazocine				
			MDZ + Pentazocine				
Jalowiecki et al. (2005a)	19/21/24	I.V. infusion (1 μg/kg) over 15 min before procedure, and then infusion (0.2 μg/kg/h)	DEX	CS	I–II	Fentanyl	5, 7, 8
			Meperidine + MDZ				
			Fentanyl				
Lee et al. (2015)	40/40	Loading dose (1 μg/kg) I.V. in 10 min before procedure, and then continuous infusion (0.4 μg/kg/h)	DEX MDZ	ESD	I–II	Pethidine and MDZ	2–5, 6, 7
Sethi et al. (2014)	30/30	Loading dose (1 μg/kg) I.V. over 10 min, and then (0.5 μg/kg/h) infusion	Fentanyl + DEX	ERCP	I–II	PF	2, 9
			Fentanyl + MDZ				
Samson et al. (2014)	30/30/30	Loading dose (1 μg/kg) infusion over 10 min, and then (0.5 μg/kg/h) continuous infusion	DEX	EGD	I–II	Fentanyl	5, 7, 8
			MDZ				
			PF				
Abbas et al. (2017)	25/25/25	Bolus 5 mL volume of normal saline containing (0.5 μg/kg)	PF + DEX	EGD	I–II	PF	5
			PF + Saline				
			PF + Ketamine				
Ahmed et al. (2020)	50/50	Bolus (1 ug/kg) over 10 min, and then continuous infusion (0.5 ug/kg/h) during procedure	DEX	CS	I–II	N.A.	4, 6–8
			PF				
Nonaka et al. (2018)	29/29	Continuous infusion (6 μg/kg/h) for 10min to induce sedation, and then continuous infusion (0.5 μg/kg/h)	PF + DEX	ESD	I–II	PF	3, 5, 6–8
			PF				
Kilic et al. (2011)	25/25	Loading dose (1 μg/kg/h), and then 0.2–0.7-µg/kg/h infusion	DEX	ERCP	I-II	N.A.	2, 9
			Fentanyl				
			MDZ				
Ramkiran et al. (2015)	24/24/24	Bolus (1 μg/kg), and then continuously infusion (0.5 μg/kg/h)	PF + DEX	ERCP	I–III	PF	5
			PF + Ketamine				
			PF + Saline				
Koruk et al. (2020)	20/20	I.V. injected (1 μg/kg) in 10 min before PF administration	PF + DEX	ERCP	I–III	PF	5, 7
			PF + MDZ				
Pushkarna et al. (2019)	30/30	Loading dose (1 μg/kg) I.V. over 10 min, and then 0.5 μg/kg/h infusion	PF + DEX	ERCP	II-III	PF	2, 3, 5
			PF + MDZ				
Amri et al. (2018)	40/40	Infusion (1 μg/kg) 10 min before procedure, and then (0.5 μg/kg/h) during procedure	DEX	CS	I-II	PF	3, 4
			Fentanyl				
Karanth et al. (2018)	30/30	Loading dose (1 μg/kg) I.V. over 10 min, and then continuous infusion (0.2–0.8 μg/kg/h) during procedure	DEX	CS	I-II	Fentanyl	7, 8
			Fentanyl				
Kavousi et al. (2021)	35/35	I.V. (1µ/kg) 1 min before procedure	DEX	CS	I-II	Fentanyl	5
			PF				
Algharabawy et al. (2021)	35/35	I.V. (1 μg/kg) over 10 min, and then (0.1 μg/kg/h) for maintenance	Ketamine + DEX	EGD	II-III	Ketamine	1, 2, 5, 6–8
			Ketamine + PF				
Pourfakhr et al. (2019)	35/35	Infusion (1 μg/kg) 1 min before procedure	Fentanyl + DEX	CS	I-II	Fentanyl	5
			Fentanyl + Ketamine				
Srivastava et al. (2018)	35/35/35	Loading dose (1 μg/kg) over 10 min	PF + DEX	ERCP	I-II	PF	2–5, 7, 9
			PF + Fentanyl				
			PF + MDZ				
Eldesuky Ali Hassan (2015)	25/25	Loading dose (1 μg/kg) I.V. over 10 min, and then infusion (0.5 μg/kg/h)	DEX	ERCP	I-II	PF	2, 5, 6, 9
			Ketamine + PF				
Abdalla et al. (2015)	30/30	Loading dose I.V. (1 μg/kg) over 15 min, and then infusion (0.5 μg/kg/h) during procedure	PF + DEX	ERCP	I-II	PF	5
			PF + Ketamine				
Mukhopadhyay et al. (2015)	15/15/15	Infusion (1 μg/kg) for 7–10 min, and then (0.2–0.5 μg/kg/h) for ≥30 min	Ketamine + PF + MDZ + Pentazocine + DEX	ERCP	I–III	PF	3–5, 6
			PF + MDZ				
			Ketamine + PF + MDZ + Pentazocine				
Koksal et al. (2014)	40/40	Loading dose infusion (0.5 μg/kg/10 min), and then (0.2 μg/kg/min)	Ketamine + DEX	EGD	I-II	PF	1, 5, 6, 8
			Ketamine + Remifentanil				

Abbreviations: EGD, upper gastrointestinal endoscopy; EOPD, endoscopic oesophageal procedures; CS, colonoscopy; ERCP, endoscopic retrograde cholangiopancreatography; ESD, endoscopic submucosal dissection; ESOD, esophagogastroduodenal endoscopy; I.V., intravenous; N.A., not available; DEX, dexmedetomidine; PF, propofol; MDZ, midazolam; ASA: american society of anesthesiologists; 1: Ramsay Sedation Scale (RSS) score; 2: Body movements or gagging; 3: Endoscopist satisfaction level; 4: Patient satisfaction level; 5: Reduction in other sedatives requirements; 6: Hypoxia; 7: Hypotension; 8: Bradycardia; 9: Cough.

### Risk-of-bias assessment

The risk of bias assessment for included RCTs is summarized in Table 2 and Supplementary Figure S1. In general, the included trials had a low risk of bias, apart from several studies. Four RCTs did not describe the specific methods used for random sequence generation. Allocation concealment was unclear in 16 RCTs. Blinding of participants was unclear in 2 RCTs, and 7 RCTs were inadequate blinding of participants (single-blind). Six RCTs were deemed to be of high risk of bias due to blinding of outcomes assessment was not carried out. Adequate assessment of incomplete outcomes was reported in all 40 RCTs. The study by Wu *et al.* was a retrospective randomized study and therefore had a potential bias in selective reporting and other biases. All other RCTs avoided selective outcome reporting and were free from other biases.

**TABLE 2:** Risk of bias evaluation of the included studies.

Study	Random sequence generation	Allocation concealment	Blinding in performance	Blinding of outcome assessment	Incomplete outcome data	Selective reporting	Other bias
Cheung et al. (2015)	Low	Low	Low	Low	low	Low	Low
Yin et al. (2019)	Low	Low	Low	Low	Low	Low	Low
Eberl et al. (2016)	Low	Unclear	Low	Low	Low	Low	Low
Edokpolo et al. (2019)	Low	Low	Low	Low	Low	Low	Low
Goyal et al. (2016)	Low	Low	Low	Low	Low	Low	Low
Ashikari et al. (2021)	Low	Low	Low	Low	Low	Low	Low
Wu et al. (2014)	Low	Unclear	Unclear	High	Low	Unclear	Unclear
Mazanikov et al. (2013)	Low	Unclear	Low	Low	Low	Low	Low
Kinugasa et al. (2018)	Unclear	Unclear	Low	Low	Low	Low	Low
Lu et al. (2018)	Low	Low	Unclear	Low	Low	Low	Low
Demiraran et al. (2007)	Low	Unclear	Low	High	Low	Low	Low
Akarsu Ayazoğlu et al. (2013)	Unclear	Unclear	Unclear	High	Low	Low	Low
Aminnejad et al. (2022)	Low	Unclear	Low	Low	Low	Low	Low
Vázquez-Reta et al. (2011)	Low	Low	Low	Low	Low	Low	Low
Wu et al. (2015)	Low	Low	Low	Low	Low	Low	Low
Chen et al. (2022)	Low	Low	Unclear	Low	Low	Low	Low
Kim et al. (2015)	Low	Low	Unclear	Low	Low	Low	Low
Lee et al. (2014)	Low	Low	Low	Low	Low	Low	Low
Takimoto et al. (2011)	Low	Low	Low	Low	Low	Low	Low
Jalowiecki et al. (2005a)	Low	Unclear	Unclear	High	Low	Low	Low

### Meta-analysis results

#### Primary outcomes

##### Ramsay sedation scale (RSS) score

**FIGURE 2:**
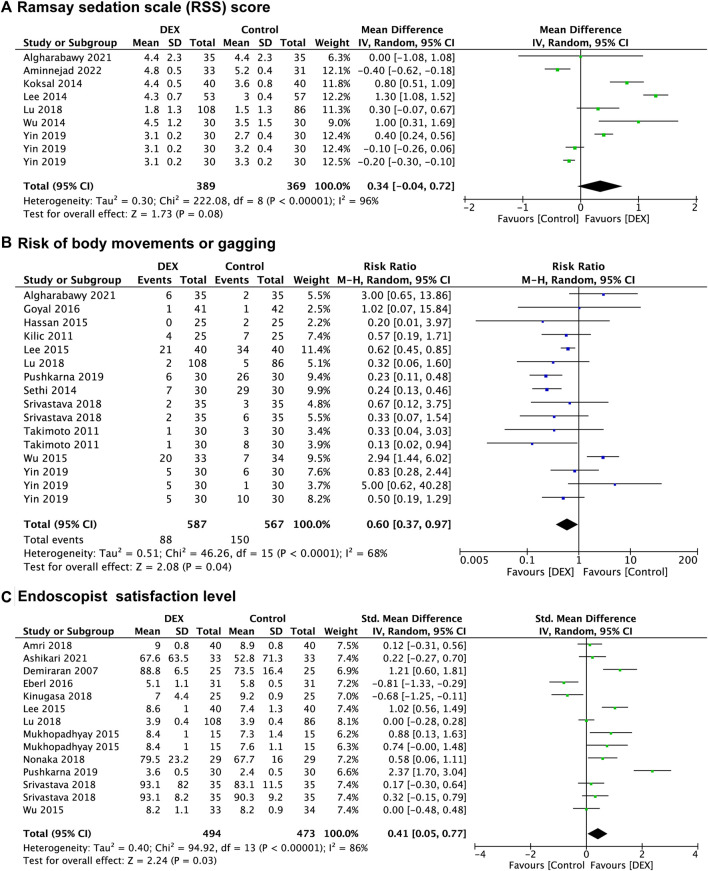
Forest plot depicting Ramsay sedation scale (RSS) score **(A)**, risk of body movements or gagging **(B)**, and endoscopist satisfaction level **(C)**. Boxes represent mean differences, and the line across each box represents respective 95% CI. CI, confidence interval; DEX, dexmedetomidine; SD, standard deviation.

Seven studies (Algharabawy et al., 2021; Aminnejad et al., 2022; Koksal et al., 2014; B. S. Lee et al., 2014; Lu et al., 2018; Wu et al., 2014; Yin et al., 2019) reported the RSS score of patients, with 369 patients in the control group vs. 389 patients in the DEX group. There was no significant difference between the DEX group and the control group in RSS score of patients (WMD: 0.34; 95% CI: −0.04 to 0.72; *p* = 0.08; I^2^ = 96%) (Figure 2A).

In the subgroup analysis (Supplementary Figure S2), there was no significant difference in RSS score of patients between the DEX group and the saline group (*p* = 0.06), the midazolam group (*p* = 0.09), and the opioids group (*p* = 0.45). Subgroup analysis also indicated that there was no significant difference in RSS score of patients between the DEX group and the control group in both the non-advanced endoscopic procedures (*p* = 0.29) and the advanced endoscopic procedures (*p* = 0.10).

##### Body movements or gagging

Twelve studies (Kilic et al., 2011; Takimoto et al., 2011; Eldesuky Ali Hassan, 2015; Lee et al., 2015; Wu et al., 2015; Goyal et al., 2016; Lu et al., 2018; Srivastava et al., 2018; Pushkarna et al., 2019; Yin et al., 2019; Algharabawy et al., 2021) recorded the prevalence of body movements or gagging, with 567 patients in the control group vs. 587 patients in the DEX group. The prevalence of body movements or gagging was significantly decreased in the DEX group (RR: 0.60; 95% CI: 0.37 to 0.97; *p* = 0.04; I^2^ = 68%) compared with the control group (Figure 2B).

In the subgroup analysis (Supplementary Figure S3), the prevalence of body movements or gagging was significantly decreased in the DEX group compared with the midazolam group (RR: 0.35; 95% CI: 0.20 to 0.59; *p* < 0.0001; I^2^ = 59%), but not in the propofol group (*p* = 0.18) and the opioids group (*p* = 0.60). There was a significant decrease in the prevalence of body movements or gagging between the DEX group and the control group in the advanced endoscopic procedures (RR: 0.37; 95% CI: 0.25 to 0.57; *p* < 0.00001; I^2^ = 35%), but not in the non-advanced endoscopic procedures (*p* = 0.32).

##### Endoscopist satisfaction level

Twelve studies (Demiraran et al., 2007; Abdalla et al., 2015; Lee et al., 2015; Wu et al., 2015; Eberl et al., 2016; Amri et al., 2018; Kinugasa et al., 2018; Lu et al., 2018; Nonaka et al., 2018; Srivastava et al., 2018; Pushkarna et al., 2019; Ashikari et al., 2021) evaluated endoscopist satisfaction level, and data from 967 patients were recorded, of which 473 were in the control group and 494 were in the DEX group. There was a significant increase in endoscopist satisfaction level in the DEX group (SMD: 0.41; 95% CI: 0.05 to 0.77; *p* = 0.03; I^2^ = 86%) compared with the control group (Figure 2C).

In the subgroup analysis (Supplementary Figure S4), endoscopist satisfaction level was significantly increased in the DEX group (SMD: 0.92; 95% CI: 0.15 to 1.69; *p* = 0.02; I^2^ = 93%) compared with the midazolam group, but no significant differences were found in the propofol group (*p* = 0.33) and the opioids group (*p* = 0.19). There was no significant difference between groups in the non-advanced endoscopic procedures (*p* = 0.23), and the advanced endoscopic procedures (*p* = 0.07). For the subgroup analysis of different scoring systems, the pooled results suggested that there was no significant difference between the DEX group and the control group in both the Numeric rating scores (*p* = 0.32) and the VAS scores (0–10) (*p* = 0.19), however, endoscopist satisfaction level of DEX group was higher than the control group in the VAS scores (0–100) (SMD: 9.63; 95% CI: 2.15 to 17.12; *p* = 0.01; I^2^ = 63%).

##### Patient satisfaction level

**FIGURE 3:**
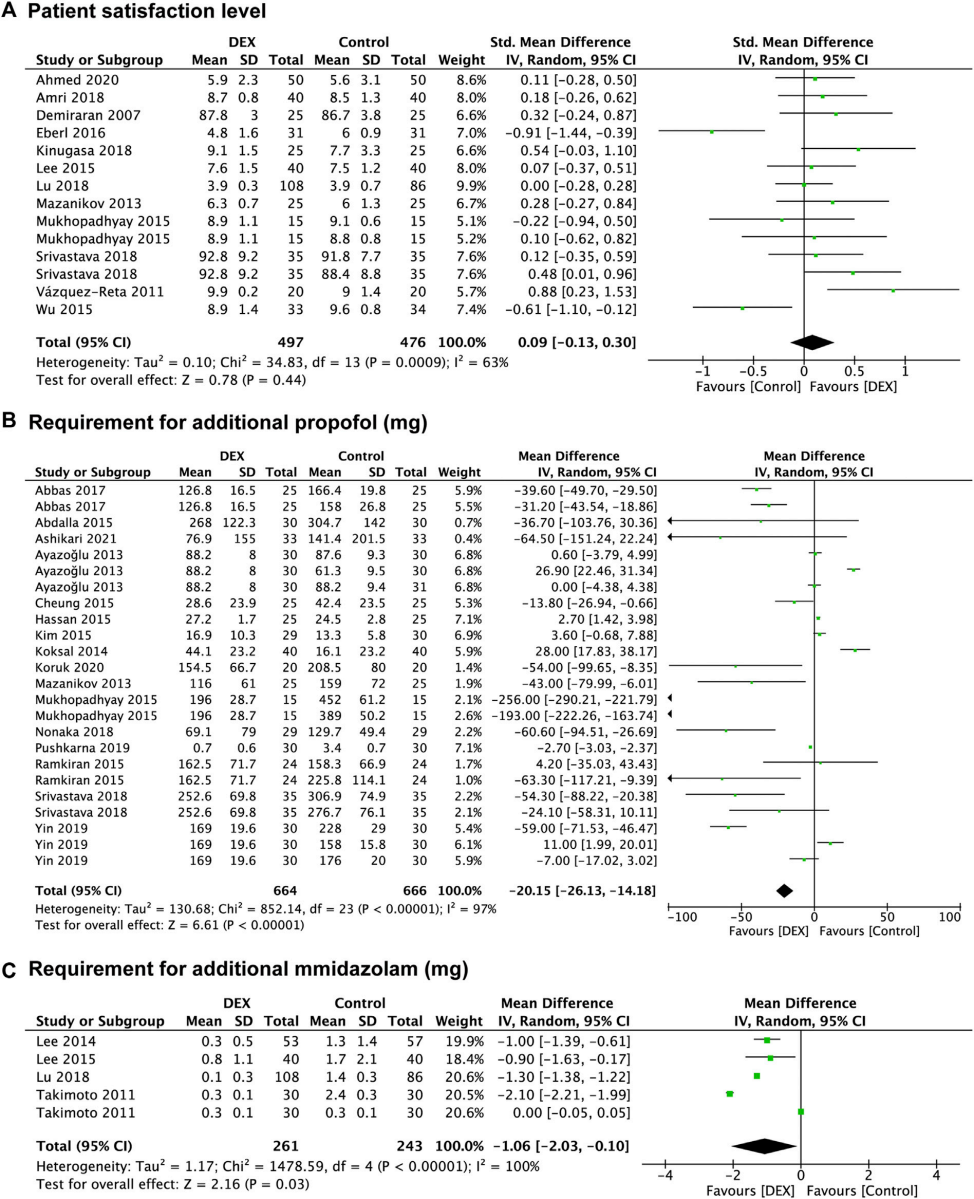
Forest plot depicting patient satisfaction level **(A)**, the requirement for additional propofol **(B)** and midazolam **(C)**. Boxes represent mean differences, and the line across each box represents respective 95% CI. CI, confidence interval; DEX, dexmedetomidine; SD, standard deviation.

Twelve studies (Demiraran et al., 2007; Vázquez-Reta et al., 2011; Mazanikov et al., 2013; Lee et al., 2015; Mukhopadhyay et al., 2015; Wu et al., 2015; Eberl et al., 2016; Amri et al., 2018; Kinugasa et al., 2018; Lu et al., 2018; Srivastava et al., 2018; Ahmed et al., 2020) evaluated patient satisfaction level, and data from 973 patients were recorded, of which 476 were in the control group and 497 were in the DEX group. Compared with the control group, the pooled SMD of patient satisfaction level in the DEX group was 0.09 (95% CI: −0.13 to 0.30; *p* = 0.44; I^2^ = 63%), indicating no significant difference between the two groups (Figure 3A).

In the subgroup analysis (Supplementary Figure S5), no significant difference was found in terms of patient satisfaction level between the DEX group and the propofol group (*p* = 0.16), midazolam group (*p* = 0.06), and opioids group (*p* = 0.35). Both the non-advanced endoscopic procedures (*p* = 0.49) and the advanced endoscopic procedures (*p* = 0.68) had no significant difference between the DEX group and the control group. For the subgroup analysis of different scoring systems, the pooled results indicated that there was no significant difference in patient satisfaction level between the DEX group and the control group in the Seven-step numeric range Likert scale (*p* = 0.68) or the VAS scores (0–10) (*p* = 0.50) or the VAS scores (0–100) (*p* = 0.06).

##### Reduction in other sedatives requirements

**FIGURE 4:**
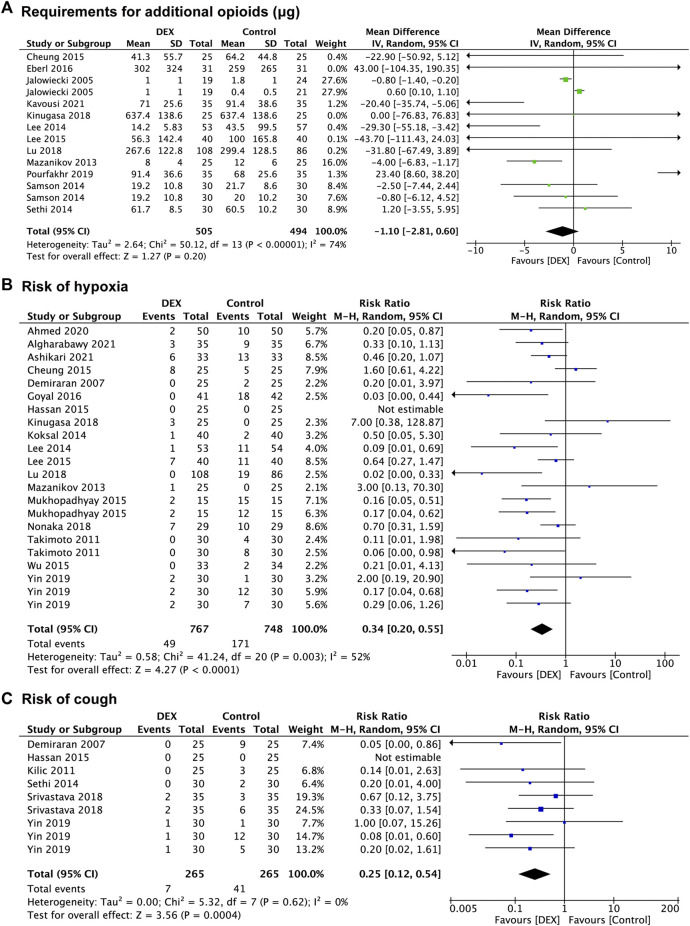
Forest plot depicting the requirement for additional opioids **(A)**, risk of hypoxia **(B)**, and risk of cough **(C)**. Boxes represent mean differences, and the line across each box represents respective 95% CI. CI, confidence interval; DEX, dexmedetomidine; SD, standard deviation.

Twenty-seven studies provided data regarding the reduction in other sedatives requirements, which was measured in 1,256 patients in the control group and 1,287 patients in the DEX group. The requirement for additional propofol was significantly reduced in the DEX group (WMD: −20.15; 95% CI: −26.13 to −14.18; *p* < 0.00001; I^2^ = 97%) compared with the control group (Figure 3B), and data from 16 studies (Akarsu Ayazoğlu et al., 2013; Mazanikov et al., 2013; Koksal et al., 2014; Abdalla et al., 2015; Cheung et al., 2015; Eldesuky Ali Hassan, 2015; Kim et al., 2015; Mukhopadhyay et al., 2015; Ramkiran et al., 2015; Abbas et al., 2017; Nonaka et al., 2018; Srivastava et al., 2018; Pushkarna et al., 2019; Yin et al., 2019; Koruk et al., 2020; Ashikari et al., 2021) with 24 comparison groups. There was also a significant reduction of the requirement for additional midazolam in the DEX group (WMD: −1.06; 95% CI: −2.03 to −0.10; *p* = 0.03; I^2^ = 100%) compared with the control group (Figure 3C), and data from 3 studies (Takimoto et al., 2011; Lee et al., 2014; Lee et al., 2015) with 5 comparison groups. We found no significant difference in the requirement for additional opioids between the two groups (*p* = 0.20) (Figure 4A), and data from 12 studies (Jalowiecki et al., 2005a; Mazanikov et al., 2013; Lee et al., 2014; Samson et al., 2014; Sethi et al., 2014; Cheung et al., 2015; Lee et al., 2015; Eberl et al., 2016; Kinugasa et al., 2018; Lu et al., 2018; Pourfakhr et al., 2019; Kavousi et al., 2021) with 14 comparison groups.

In the subgroup analysis of opioids consumption, the requirement for pethidine was significantly decreased in the DEX group (WMD: −28.33; 95% CI: −51.39 to −5.27; *p* = 0.02; I^2^ = 0%) compared with the control group, however, no significant difference was found between the two groups in the requirement for fentanyl (*p* = 0.83) and alfentanil (*p* = 0.17). The subgroup analysis of the requirement for opioids between the two groups identified no significant difference in both the non-advanced endoscopic procedures (*p* = 0.64) and the advanced endoscopic procedures (*p* = 0.19) (Supplementary Figure S6). The subgroup analysis of the requirement for propofol showed that the DEX group had significantly lower requirements than the control group in the advanced endoscopic procedures (WMD: −36.10; 95% CI: −44.74 to −27.45; *p* < 0.00001; I^2^ = 97%), but not in the non-advanced endoscopic procedures (*p* = 0.27). There was no significant difference in the requirement for midazolam between the DEX group and the control group in the advanced endoscopic procedures (*p* = 0.16) (Supplementary Figure S7).

### Secondary outcomes

#### Adverse events

##### Hypoxia

Eighteen studies (Demiraran et al., 2007; Takimoto et al., 2011; Mazanikov et al., 2013; Koksal et al., 2014; Lee et al., 2014; Cheung et al., 2015; Eldesuky Ali Hassan, 2015; Lee et al., 2015; Mukhopadhyay et al., 2015; Wu et al., 2015; Goyal et al., 2016; Kinugasa et al., 2018; Lu et al., 2018; Nonaka et al., 2018; Yin et al., 2019; Ahmed et al., 2020; Algharabawy et al., 2021; Ashikari et al., 2021) reported data concerning the risk of hypoxia, which was observed in 748 patients in the control group and 767 patients in the DEX group. The risk of hypoxia was significantly decreased in the DEX group (RR:0.34; 95% CI: 0.20 to 0.55; *p* < 0.0001; I^2^ = 52%) compared with the control group (Figure 4B).

In the subgroup analysis, the DEX group carried a lower risk of hypoxia (RR: 0.23; 95% CI: 0.10 to 0.54; *p* = 0.0007; I^2^ = 0%) than the propofol group, however, the results indicated no significant difference between the DEX group and saline group (*p* = 0.18), and the midazolam group (*p* = 0.07), and the opioids group (*p* = 0.09). Compared with the control group, the pooled RR of hypoxia when using DEX was 0.42 (95% CI: 0.21 to 0.85; *p* = 0.01; I^2^ = 36%) in the non-advanced endoscopic procedures and 0.28 (95% CI: 0.13 to 0.58; *p* = 0.0006; I^2^ = 61%) in the non-advanced endoscopic procedures, showing a significantly lower risk of hypoxia in the DEX group (Supplementary Figure S8). When the definition of hypoxia was SpO_2_ < 90%, the risk of hypoxia was decreased in the DEX group (RR: 0.28; 95% CI: 0.15 to 0.53; *p* < 0.0001; I^2^ = 40%) compared with the control group, but no significant difference was found when the definition of hypoxia was SpO_2_ < 94% (*p* = 0.06) (Supplementary Figure S8).

##### Hypotension

**FIGURE 5:**
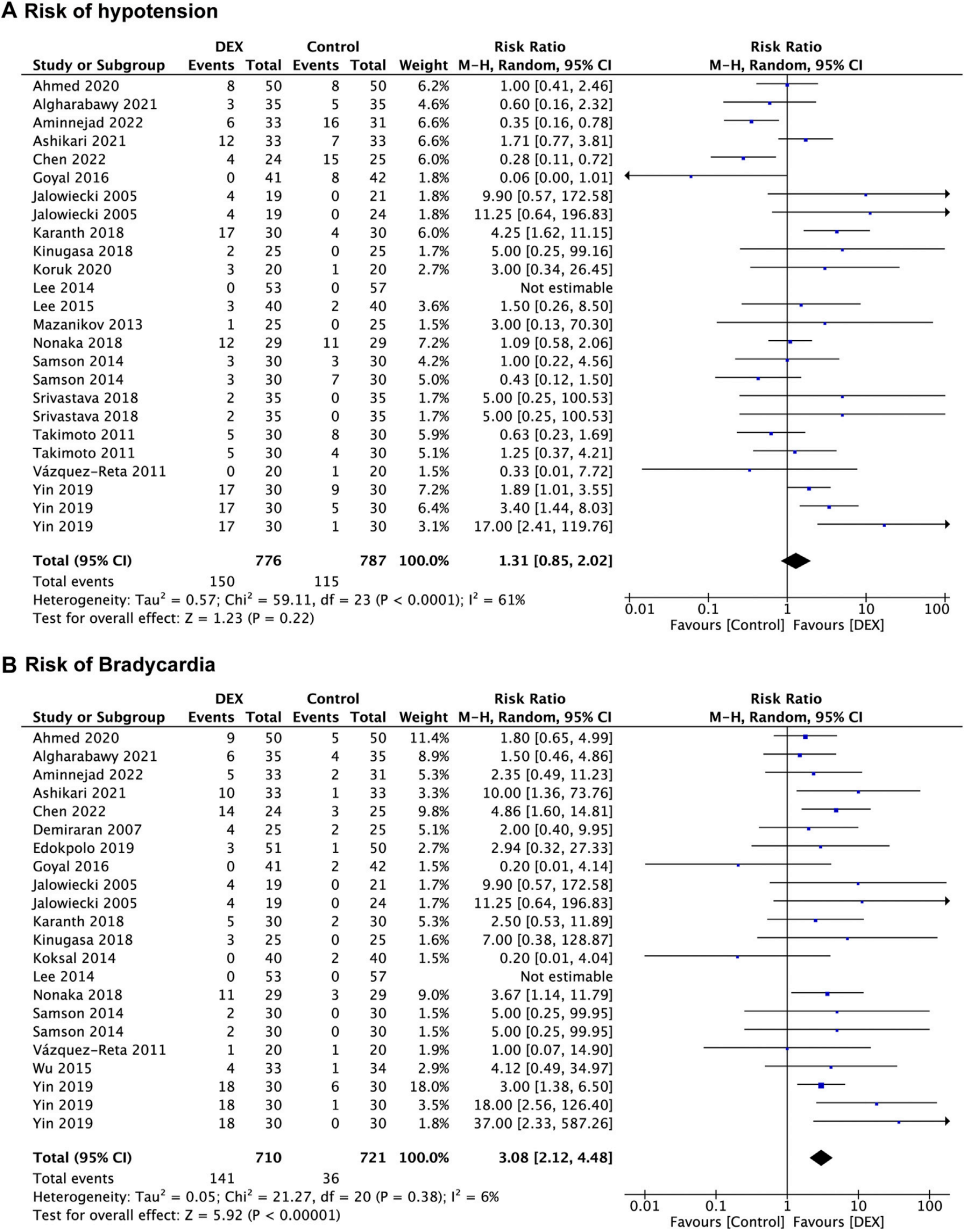
Forest plot depicting risk of hypotension **(A)**, and risk of bradycardia **(B)**. Boxes represent mean differences, and the line across each box represents respective 95% CI. CI, confidence interval; DEX, dexmedetomidine; SD, standard deviation.

Nineteen studies (Jalowiecki et al., 2005a; Takimoto et al., 2011; Vázquez-Reta et al., 2011; Mazanikov et al., 2013; Lee et al., 2014; Samson et al., 2014; Lee et al., 2015; Goyal et al., 2016; Karanth et al., 2018; Kinugasa et al., 2018; Nonaka et al., 2018; Srivastava et al., 2018; Yin et al., 2019; Ahmed et al., 2020; Koruk et al., 2020; Algharabawy et al., 2021; Ashikari et al., 2021; Aminnejad et al., 2022; Chen et al., 2022) recorded the risk of hypotension, with 787 patients in the control group vs. 776 patients in the DEX group. There was no significant difference in risk of hypotension between the DEX group (RR: 1.31; 95% CI: 0.85 to 2.02; *p* = 0.22; I^2^ = 61%) and the control group (Figure 5A).

In the subgroup analysis, the risk of hypotension in the DEX group was higher than saline group (RR: 1.84; 95% CI: 1.13 to 3.00; *p* = 0.01; I^2^ = 0%) and the opioids group (RR: 3.99; 95% CI: 2.16 to 7.37; *p* < 0.00001; I^2^ = 0%), whereas the DEX group had a lower risk of hypotension than the propofol group (RR: 0.55; 95% CI: 0.34 to 0.88; *p* = 0.01; I^2^ = 0%), and no significant difference was found between the DEX group and the midazolam group (*p* = 0.40). Pooled results revealed that both the non-advanced endoscopic procedures (*p* = 0.19) and the advanced endoscopic procedures (*p* = 0.87) had no significant difference in hypotension risk between the control group and the DEX group (Supplementary Figure S9).

##### Bradycardia

Eighteen studies (Jalowiecki et al., 2005a; Demiraran et al., 2007; Vázquez-Reta et al., 2011; Koksal et al., 2014; Lee et al., 2014; Samson et al., 2014; Wu et al., 2015; Goyal et al., 2016; Karanth et al., 2018; Kinugasa et al., 2018; Nonaka et al., 2018; Edokpolo et al., 2019; Yin et al., 2019; Ahmed et al., 2020; Algharabawy et al., 2021; Ashikari et al., 2021; Aminnejad et al., 2022; Chen et al., 2022) reported data concerning the risk of bradycardia, which was observed in 721 patients in the control group and 710 patients in the DEX group. There was a significant increase in bradycardia risk in the DEX group (RR: 3.08; 95% CI: 2.12 to 4.48; *p* < 0.00001; I^2^ = 6%) compared with the control group (Figure 5B).

In the subgroup analysis (Supplementary Figure S10), compared with the saline group (RR: 3.45; 95% CI: 1.74 to 6.85; *p* = 0.0004; I^2^ = 0%) and the propofol group (RR: 2.53; 95% CI: 1.40 to 4.59; *p* = 0.002; I^2^ = 0%), the DEX group had a higher risk of bradycardia, but no significant difference was found between the midazolam group (*p* = 0.27), the opioids group (*p* = 0.14) and the DEX group. The results of subgroup analysis displayed a higher risk of bradycardia in the DEX group than in the control group in both non-advanced endoscopic procedures (RR: 2.80; 95% CI: 1.82 to 4.32; *p* < 0.00001; I^2^ = 6%) and advanced endoscopic procedures (RR: 4.04; 95% CI: 1.77 to 9.19; *p* = 0.0009; I^2^ = 17%).

##### Cough

Six studies (Demiraran et al., 2007; Kilic et al., 2011; Sethi et al., 2014; Eldesuky Ali Hassan, 2015; Srivastava et al., 2018; Yin et al., 2019) recorded the risk of cough, with 265 patients in the control group vs. 265 patients in the DEX group. The risk of cough was significantly decreased in the DEX group (RR: 0.25; 95% CI: 0.12 to 0.54; *p* = 0.0004; I^2^ = 0%) compared with the control group (Figure 4C).

In the subgroup analysis (Supplementary Figure S11), the risk of cough was lower in the DEX group (RR: 0.20; 95% CI: 0.07 to 0.62; *p* = 0.005; I^2^ = 0%) than in the midazolam group, but no difference in the opioids group (*p* = 0.19). The risk of cough in the DEX group was significantly decreased in the DEX group compared with the control group in both the non-advanced endoscopic procedures (RR: 0.16; 95% CI: 0.05 to 0.50; *p* = 0.002; I^2^ = 0%) and the advanced endoscopic procedures (RR: 0.36; 95% CI: 0.13 to 0.98; *p* = 0.05; I^2^ = 0%).

##### Sensitivity analysis and publication bias

A sensitivity analysis was performed to reduce the potential bias (Supplementary Figures S1–S11), and no single trial significantly affected the overall results of most outcomes in this meta-analysis, except for the requirement for additional propofol in which three studies (Eldesuky Ali Hassan, 2015; Mukhopadhyay et al., 2015; Pushkarna et al., 2019) with a high risk of bias but did not alter the findings. Potential publication bias was evaluated graphically using funnel plot asymmetry, and funnel plot asymmetry was measured by Egger’s regression test. No asymmetry was demonstrated by a visual indication of the funnel plots, and Egger’s regression test suggested no significant asymmetry of the funnel plots in all outcomes (*p* > 0.05) (Supplementary Table S1), indicating no evidence of significant publication bias in this meta-analysis.

## Discussion

**FIGURE 6:**
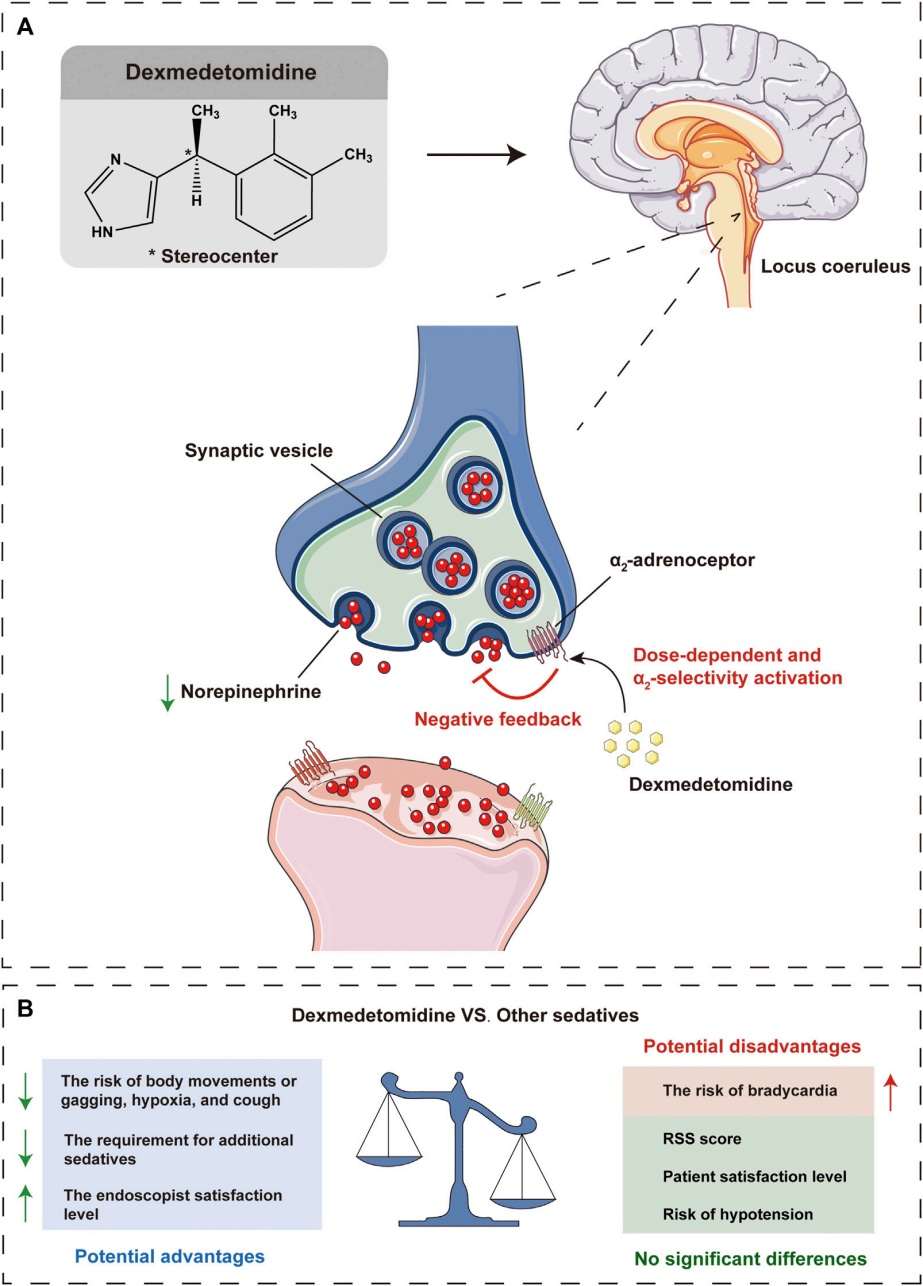
Schematic illustration that dexmedetomidine inhibits norepinephrine release, resulting in a reduction of excitation, especially in the locus coeruleus, which mediates the sedative and antinociceptive effects **(A)**. Potential advantages and disadvantages of dexmedetomidine compared with other sedatives in gastrointestinal endoscopic procedures **(B)**. VS, versus; RSS, Ramsay sedation scale.

These data suggest that the primary outcomes of sedation level of DEX are comparable to other sedatives, with similar RSS score and patient satisfaction level, and even better in some clinical outcomes, with decreased risk of body movements or gagging and reduced additional requirement for other sedatives and increased endoscopist satisfaction level. In terms of secondary outcomes of adverse events, DEX may benefit patients in some clinical outcomes (reduced risk of hypoxia and cough), with no significant difference in the risk of hypotension, while there may be potential drawbacks in other outcomes (increased risk of bradycardia) (Figure 6B).

In terms of sedation level, our results indicated that DEX is comparable to other sedatives with similar RSS score and patient satisfaction level, but a higher endoscopist satisfaction level. An admirable property of DEX is arousable sedation mimicking natural sleep, which is indicative of the potential of DEX for an easy transition from sleep to wakefulness potential of DEX, thus allowing patients to be cooperative and communicative when stimulated which may be the reason for the higher satisfaction level of endoscopists and also make the endoscopy a more smooth procedure (Wijeysundera et al., 2009; Liu X et al., 2021). The results of sedation level in the present analysis are conflicted with several previous studies comparing DEX with propofol or midazolam (Nishizawa et al., 2015; Zhang et al., 2016; Nishizawa et al., 2017), which may be related to the inclusion of multiple different comparators, and we will discuss these outcomes in subgroup analysis for different comparators. Another desirable property of DEX is the additional opioids and sedatives sparing effect (Akarsu Ayazoğlu et al., 2013; Wang and Shi, 2017; Nonaka et al., 2018), which may be associated with the synergistic interactions of DEX with other sedatives due to its different sedative mechanism than conventional sedatives. In our meta-analysis, additional consumption of propofol and midazolam was significantly reduced with DEX sedatives, and there was a trend toward a reduction in opioids consumption although no statistical difference was found, however, a subgroup analysis confined to the 3 studies showed a lower consumption of pethidine when using DEX sedative. This founding revealed that DEX can provide adequate conscious sedation during gastrointestinal endoscopic procedures while reducing the requirement for other additional sedatives, with significant clinical implications for decreasing the dose of each drug and minimizing individual adverse side effects.

As a highly selective α2-adrenoceptor agonist, DEX acts primarily on the central pre-and postsynaptic α2-receptors in the locus coeruleus which gives it a unique sedative activity that differs from conventional sedatives (Figure 6A) (Hoy and Keating, 2011; Liu X et al., 2021). For example, DEX exhibits minimal respiratory depressive effects and lower physiologic stress response to surgical stimulation than GABA receptor agonists such as propofol and the benzodiazepines (Riker et al., 2009; Candiotti et al., 2010; Akarsu Ayazoğlu et al., 2013). The results of this current meta-analysis suggested that DEX sedation had statistically lower hypoxia risk and body movements or gagging rate, which was consistent with the conclusions of the previous studies (Nishizawa et al., 2015; Liu W et al., 2021), and also with the pharmacological characteristics of DEX, but contradicted by others (no significant difference was found) (Nishizawa et al., 2015; Nishizawa et al., 2017). This is a potentially critical finding, as involuntary patient body movements or gagging can severely interfere with endoscopic interventions and may increase risk of adverse events. Furthermore, DEX sedation with a lower risk of hypoxia may result in a more stable respiratory system, which may be beneficial in patients with a history of respiratory disease.

Bradycardia and hypotension are two other common cardiorespiratory complications of DEX besides hypoxia, both usually resolve without intervention (Hoy and Keating, 2011). As the mainly hemodynamic effects and known side effects of DEX, dose-dependent bradycardia and hypotension are caused by its endogenous catecholamine reduction, peripheral vasoconstrictive, sympatholytic, and baroreflex-mediated parasympathetic activation properties (Jalowiecki et al., 2005b; Carollo et al., 2008; Weerink et al., 2017), which are associated with the activation of α2-adrenoceptor agonist and imidazoline-preferring receptors in the ventrolateral medulla and solitarius nucleus tract (Hayashi et al., 1995). This current meta-analysis found that DEX significantly increased the risk of bradycardia, although we pooled the data with different definitions of bradycardia, the heterogeneity was as low as 6%. Although previous studies have not reached consistent conclusions about risk of bradycardia associated with DEX (Nishizawa et al., 2015; Zhang et al., 2016; Nishizawa et al., 2017), we suggest that DEX should be used with caution in patients diagnosed with severe sinus bradycardia or heart block. During gastrointestinal endoscopic procedures, bradycardia could be managed with atropine or butylscopolamine bromide. However, no statistically significant difference was found in the risk of hypotension. It is important to note that we appear to have identified a source of heterogeneity in the risk of hypotension, which was reduced to 0% for all subgroups by subgroup analysis with different comparators, but there were considerable variations in results between subgroups which would be discussed below. Therefore, a complicated method of DEX administration in somewhat, including a loading dose that should be given over no less than 10 min and a maintenance dose with appropriate infusion velocity, may achieve favorable cardiovascular stability (Schaffrath et al., 2004; Nishizawa et al., 2015).

Perioperative DEX administration has been suggested to reduce the occurrence of cough in many surgeries since its property of mitigating airway reflexes (Kim et al., 2013; Aouad et al., 2019). Perioperative coughing is highly undesirable for patients as it may prolong extubation and delay postoperative recovery, which may lead to unfavorable postoperative complications. The results and several subgroup analyses in this present meta-analysis showed that DEX significantly reduced the risk of cough, with 0% heterogeneity in all comparisons. Although no previous meta-analysis has pooled outcomes of cough risk in gastrointestinal endoscopic procedures, our findings support that DEX administration may be effective for perioperative cough prevention.

In the main analysis, we included all comparators and procedure types, most analysis results were limited by significant heterogeneity that may have influenced the validity of our results. Therefore, we performed subgroup analyses to address this issue, among which the subgroup analysis of different comparators had a more obvious effect of reducing heterogeneity. We discuss the advantages and disadvantages of DEX over other sedatives and present the results of the subgroup analysis in Table 3.

**TABLE 3:** Subgroup analysis of DEX versus different sedation comparators in the primary and secondary outcomes.

Subgroup analysis	Clinical outcomes and results
DEX VS Saline	**The primary outcomes:**
	1. RSS score (WMD: 0.85; 95% CI: −0.03 to 1.73; *p* = 0.06; I^2^ = 98%)
	**The secondary outcomes:**
	2. Hypoxia (RR: 0.47; 95% CI: 0.16 to 1.41; *p* = 0.18; I^2^ = 67%)
	3. Hypotension (RR: 1.84; 95% CI: 1.13 to 3.00; *p* = 0.01; I^2^ = 0%)
	4. Bradycardia (RR: 3.45; 95% CI: 1.74 to 6.85; *p* = 0.0004; I^2^ = 0%)
DEX VS PF	**The primary outcomes:**
	1. Body movements or gagging (RR: 2.04; 95% CI: 0.72 to 5.78; *p* = 0.18; I^2^ = 43%)
	2. Endoscopist satisfaction level (SMD: −0.40; 95% CI: −1.19 to 0.39; *p* = 0.33; I^2^ = 80%)
	3. Patient satisfaction level (SMD: −0.45; 95% CI: −1.08 to 0.17; *p* = 0.16; I^2^ = 81%)
	**The secondary outcomes:**
	4. Hypoxia (RR: 0.23; 95% CI: 0.10 to 0.54; *p* = 0.0007; I^2^ = 0%)
	5. Hypotension (RR: 0.55; 95% CI: 0.34 to 0.88; *p* = 0.01; I^2^ = 0%)
	6. Bradycardia (RR: 2.53; 95% CI: 1.40 to 4.59; *p* = 0.002; I^2^ = 0%)
DEX VS MDZ	**The primary outcomes:**
	1. RSS score (WMD: 0.59; 95% CI: −0.09 to 1.26; *p* = 0.09; I^2^ = 68%)
	2. Body movements or gagging (RR: 0.35; 95% CI: 0.20 to 0.59; *p* < 0.0001; I^2^ = 59%)
	3. Endoscopist satisfaction level (SMD: 0.92; 95% CI: 0.15 to 1.69; *p* = 0.02; I^2^ = 93%)
	4. Patient satisfaction level (SMD: 0.28; 95% CI: −0.01 to 0.57; *p* = 0.06; I^2^ = 50%)
	**The secondary outcomes:**
	5. Hypoxia (RR: 0.16; 95% CI: 0.02 to 1.19; *p* = 0.07; I^2^ = 68%)
	6. Hypotension (RR: 1.37; 95% CI: 0.66 to 2.84; *p* = 0.40; I^2^ = 0%)
	7. Bradycardia (RR: 2.02; 95% CI: 0.58 to 7.08; *p* = 0.27; I^2^ = 0%)
	8. Cough (RR: 0.20; 95% CI: 0.07 to 0.62; *p* = 0.005; I^2^ = 0%)
DEX VS Opioids	**The primary outcomes:**
	1. RSS score (WMD: 0.34; 95% CI: −0.54 to 1.22; *p* = 0.45; I^2^ = 96%)
	2. Body movements or gagging (RR: 0.78; 95% CI: 0.31 to 1.95; *p* = 0.60; I^2^ = 0%)
	3. Endoscopist satisfaction level (SMD: 0.21; 95% CI: −0.11 to 0.53; *p* = 0.19; I^2^ = 0%)
	4. Patient satisfaction level (SMD: 0.15; 95% CI: −0.17 to 0.47; *p* = 0.35; I^2^ = 0%)
	**The secondary outcomes:**
	5. Hypoxia (RR: 0.34; 95% CI: 0.10 to 1.18; *p* = 0.09; I^2^ = 0%)
	6. Hypotension (RR: 3.99; 95% CI: 2.16 to 7.37; *p* < 0.00001; I^2^ = 0%)
	7. Bradycardia (RR: 3.67; 95% CI: 0.66 to 20.51; *p* = 0.14; I^2^ = 0%)
	8. Cough (RR: 0.41; 95% CI: 0.11 to 1.55; *p* = 0.19; I^2^ = 0%)

Abbreviations: DEX, dexmedetomidine; PF, propofol; MDZ, midazolam; VS, versus; CI, confidence interval; WMD, weighted mean difference; SMD, standardized mean difference; RR, risk ratio.

Limited by the number of included studies, too few outcomes can be analysed in studies comparing DEX with saline or ketamine, and we may not be able to draw firm conclusions from these data. What we do know, however, is that DEX has both pharmacodynamic advantages of a significantly greater α 2:α 1-adrenoceptor affinity ratio and a pharmacokinetic advantage over similar α 2-adrenoceptor agonists such as ketamine and a better sedation level over saline (Liu X et al., 2021).

An interesting discovery is that although previous researchers have suggested that pooling data on outcomes of different definitions or evaluation systems may be one source of heterogeneity, however, subgroup analyses were not performed due to the small number of included studies in previous meta-analyses. In this present meta-analysis, we performed a subgroup analysis in terms of different definitions or evaluation systems of certain outcomes, and the results showed that they had little effect on heterogeneity, which deviates from the conjecture of the previous researchers.

## Limitations

Confinement to RCTs representing the highest level of evidence is a major strength of our work, however, there are also several limitations in our meta-analysis. First, the number of studies in our meta-analysis was insufficient, especially for several outcomes such as midazolam consumption and risk of cough, which may increase Type 1 error and publication bias. Of the 40 trials enrolled in our meta-analysis, only three and one trial originated from Europe and the United States, respectively, which may be another source of publication bias. Second, most of the analyses results were limited by substantial heterogeneity, however, no exact reason for the observed heterogeneity was determined although several subgroup analyses were performed, among which the subgroup analysis of different comparators had a more obvious effect of decreasing heterogeneity. It is more important to note that the association between the dose use/the mode of DEX administration and cardiac side effects such as bradycardia and hypotension was not available yet. Remarkably, synergistic sedation regimens containing DEX based on different sedatives may benefit patients more because of the admirable potential to reduce side effects, improve tolerability, and reduce the requirement for additional sedatives while providing a safe and effective sedation level. Therefore, further trials focusing on specific surgery-type-related, various dose use/the mode of DEX administration, and different synergistic sedation regimens based on DEX are warranted. Third, the extensive exclusion criteria in most trials, including American Society of Anesthesiologists (ASA) class IV or more, pregnancy, cardiovascular disease, renal or hepatic or pulmonary insufficiency, limited the applicability of the results to the general critically ill patient population, moreover, the paediatric population was excluded in this study due to the limited number of studies, however, the role of DEX as a potential sedative in the paediatric population warrants further investigation (Mason et al., 2021). Fourth, subgroup analyses were conducted in an attempt to reduce heterogeneity, but many subgroup analyses contained small studies and sample sizes and were therefore of limited value. Finally, other outcomes (such as economic cost) should be evaluated in the subgroup analysis of different sedatives to better understand their role in gastrointestinal endoscopic procedures.

## Conclusion

In summary, we did find evidence of certain advantages of DEX in gastrointestinal endoscopic procedures, whilst some potential disadvantages also exist. DEX is comparable to midazolam and propofol in maintaining light to moderate sedation and even better in some clinical outcomes during gastrointestinal endoscopic procedures, and all these indicate that DEX is a safe and effective sedative agent for gastrointestinal endoscopy and provides a more sedative option for patients undergoing gastrointestinal endoscopic procedures. However, definitive conclusions on the clinical practice of DEX in gastrointestinal endoscopic procedures may not be given due to issues of limited sample size and heterogeneity. What we did is a systematic and pooled meta-analysis of the current literature regarding DEX use in gastrointestinal endoscopic procedures, which could stimulate new research that may potentially guide future clinical sedation practices in this field. Further large, multicenter RCTs with multiple sedation protocols are warranted to enhance understanding of its pharmacological properties, patient selection, dosage, and adverse effects. Therefore, we will continue to pay attention to updating our conclusions in the future.

## Data Availability

The original contributions presented in the study are included in the article/Supplementary Material, further inquiries can be directed to the corresponding authors.

## Abbreviations

ASA, American Society of Anesthesiologists; CI, Confidence interval; CS, colonoscopy; DEX, Dexmedetomidine; EGD, upper gastrointestinal endoscopy; ERCP, Endoscopic retrograde cholangiopancreatography; ESD, Endoscopic submucosal dissection; EOPD, endoscopic oesophageal procedures; ESOD, esophagogastroduodenal endoscopy; IQR, Inter-quartile range; I.V., intravenous; SE, Mean; N.A., not available; PRISMA, Preferred Reporting Items for Systematic Review and Meta-Analysis; RSS, Ramsay sedation scale; RCT, Randomized controlled trial; RR, Risk ratios; SD, Standard deviation; SMD, Standardized mean difference; VAS, Visual analogue scale; N.A., not available; WMD, Weighted mean difference.
